# Testing international dental maturation scoring system and population-specific Demirjian versions on Saudi sub-population

**DOI:** 10.4317/jced.51373

**Published:** 2014-04-01

**Authors:** Ziad-D. Baghdadi

**Affiliations:** 1 DDS, PD, MS, PhD, MPH. Department of Preventive Dentistry, Riyadh Colleges of Dentistry and Pharmacy, Riyadh, Saudi, Arabia

## Abstract

Objectives: The purpose of this study was to test the applicability of the Demirjian method and revised versions for estimating chronological age (CA) from dental age (DA) in a sample of children. Study Design: A sample of 252 individuals of known age (4 to 14 yrs), sex (males: 125, females: 127), and ethnicity (Saudi) was collected. Each individual was aged using the original Demirjian method and revised versions, including Saudi, Kuwaiti, Belgian, and revised international curves. The differences between dental age and chronological age were analyzed using paired sample t-tests with Bonferroni corrections and multinomial regression tests at the 0.05 level of significance. Results: The results indicated an over-aging of the sample as a whole by about 10 months using Demirjian tables, 5.5 months using Kuwaiti tables, 24.7 months using Belgian tables, and 5 months using revised international tables. The sample was under-aged by 0.6 month using Saudi tables. The overall discrepancies between CA and DA were statistically significant (P < 0.0001) for all methods with the exception of Saudi curves. Conclusions: The findings suggest that the Saudi population method is most accurate on a Saudi population.

** Key words:**Age estimation, juvenile, forensic dentistry, Saudi Arabia.

## Introduction

Determining a child’s chronological age (CA) and stage of maturation is particularly important in fields such as pediatrics, orthopedics, and orthodontics, as well as in forensic and anthropological studies (1). The dental panoramic radiograph is recommended to be made periodically during the mixed dentition and adolescence to evaluate growth and development (2). As indicated in the reference manual of the American Academy of Pediatric Dentistry, Logan and Kronfeld’s work in 1933 can be considered as the first major attempt at developing a chronology for human dental and skeletal maturation and dispelling the myth that calcification of all permanent teeth begins at the same time (3). Consequently, in 1973 Demirjian *et al.* studied the dental development of a genetically homogenous French-Canadian group of children ranging in age from 2.5 to 19 years using 5,437 panoramic radiographs (4). The maturity of each mandibular tooth was evaluated individually, and developmental curves were plotted for each stage of each tooth for boys and girls.

Hegde and Sood found that the method of Demirjian overestimated the age of children from Belgaum, India, by 0.14 years for boys and 0.04 years for girls (5). The authors concluded that the method of Demirjian is accurate in predicting the age of Indians. Dental age was studied by Nykänen et al. in a sample of 261 Norwegian children (128 boys and 133 girls) and found the children were advanced in dental maturity by 1.5 to 4 months in boys and 4.5 to 7.5 in girls. Generally, 95% of the individual estimated ages were within ±2 years of the true age (6). Davidson and Rodd compared dental age with chronological age in Somali children with that of matched white Caucasian children residing in Sheffield, England (7). The mean difference between dental age and chronological age was 1.01 years for Somali boys, 0.19 for Caucasian boys, 1.22 years for Somali girls, and 0.52 years for Caucasian girls. Somali children are significantly more dentally advanced than their Caucasian peers (7). A study assessing the dental age in Saudi children aged 8.5 to 17 years found that Saudi children from Riyadh were overestimated by 0.3 years for boys and 0.4 years for girls (8). Similar results were reported on Kuwaiti children aged 3 to 14 yrs, but the overestimations were 0.71 yrs for boys and 0.67 for girls (9). These findings suggest that there is a need for population specific dental development standards based on ethnicity to improve the accuracy of dental age assessment. Similar to several authors who developed new specific population dental maturation tables and curves, Baghdadi developed new age prediction models and maturation scores for Saudi population based on the Demirjian method using multinomial functions, and he concluded that the new models need further validation studies (10). Combining data from several studies, Chaillet *et al.* established a database consisting of 9,577 dental radiographs from healthy dental patients aged 2–25 years (mainly of European origin from Canada, Europe, and Australia) (11). Derived from linear regression lines for 1-year age categories and manually smoothed, average score for age and age for score for the Chaillet database are detailed in Liversidge, who recommended their use as an international scoring system (12). It was, therefore, the aim of this cross-sectional study to test the Demirjian method of dental maturity and its modified versions on a sample of children from Saudi Arabia.

## Material and Methods

Selected from the Department of Preventive Dentistry Section of Pedodontics, Riyadh Colleges of Dentistry and Pharmacy, and two private clinics in Riyadh, Saudi Arabia, the sample consisted of 252 panoramic radiographs of the teeth from 125 boys and 127 girls (ages ranging from 4 to 14 years). Healthy, Saudi children were selected. Children with systemic diseases, which can affect development of teeth, mandibular hypodontia except third molars, and low-quality radiographs, were excluded. All radiographs were taken between January 2010 and January 2013.

The participants were divided into 10 groups according to their chronological age, which was calculated by subtracting the date of the radiograph from the date of birth taken from the child’s birth certificate. The first group, consisting of 4-years-olds, included children of ages ranging from 4.00 to 4.99, and the next group included 5-year-olds and so on.

**Figure 1:**
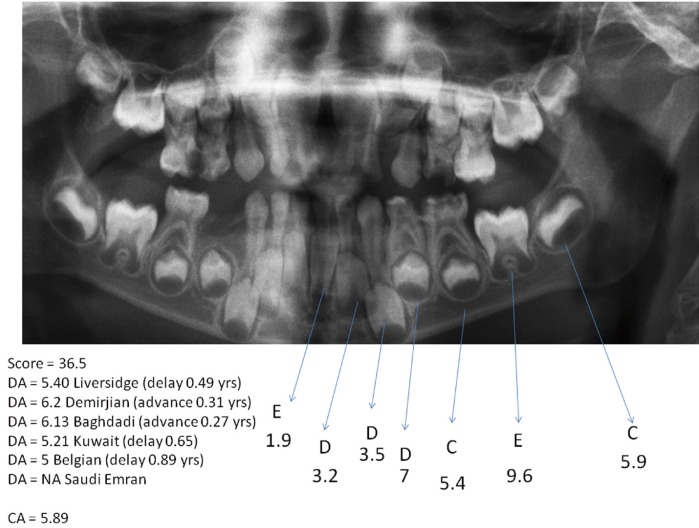
An example of how dental maturity is calculated for a boy.

Dental age assessment was performed according to the Demirjian method (4). Briefly, the development of each left permanent mandibular tooth, except the third molar, was rated on an 8-stage scale from A to H, and the criteria for the stages were given for each tooth separately. Each stage of the seven teeth was allocated a score, and the sum of the scores gave an evaluation of the child’s dental maturity, measured on a scale from 0 to 100. The score of each child was then converted to dental age by using standard Demirgian tables and revised tables specific to Saudi, Kuwaiti, and Belgian populations (4,8-10,13). The revised international tables were also used (12). An example of how dental maturity is calculated for a boy is shown in figure 1. This shows part of a panoramic dental radiograph, the seven tooth stages, their weighted values, the dental maturity score, the estimated age based on the original Demirjian tables and revised tables, and how the dental age relates to chronological age.

The stages of the seven left mandibular permanent teeth were assessed by the author without knowledge of chronological age and gender. To evaluate reproducibility, 10% of radiographs (n = 25) were randomly selected and re-assessed 2 months after the initial rating. The data were stored and analyzed using statistical software SPSS. Interclass correlation coefficient (ICC) was used for testing the intra-observer repeatability. The accuracy of the method was determined by mean difference between dental age and chronological age (DA–CA). Paired samples t-test was applied to assess the significances of the difference between dental age (DA) and chronological age (CA) for both genders and age cohorts separately. Tests with a P value less than 0.0025 can be conside-red significant according to Bonferroni correction. The correlations between dental age as a result of Demirjian maturity standards and revised versions and the chronological age and coefficients of determination were verified by the multinomial (cubic) regression analysis for girls and boys separately.

## Results

**Figure 2:**
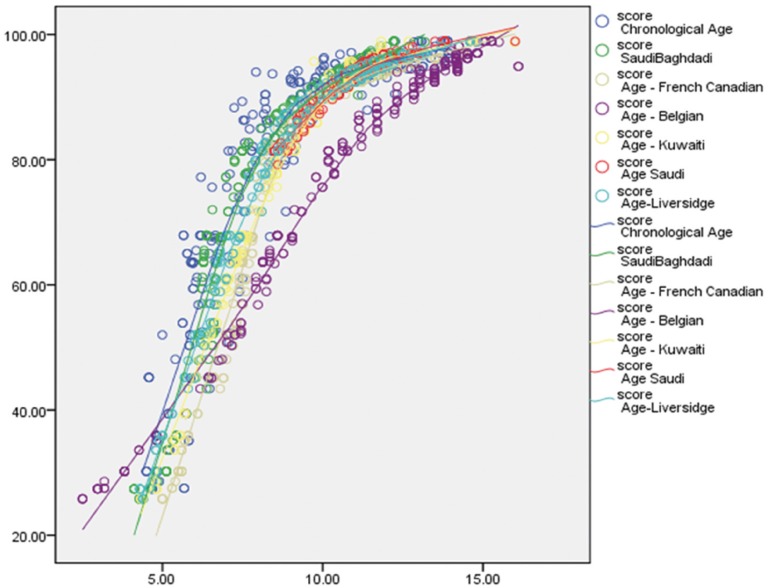
The maturation curves of the study population using the Demirjian method and revised versions.

**Figure 3:**
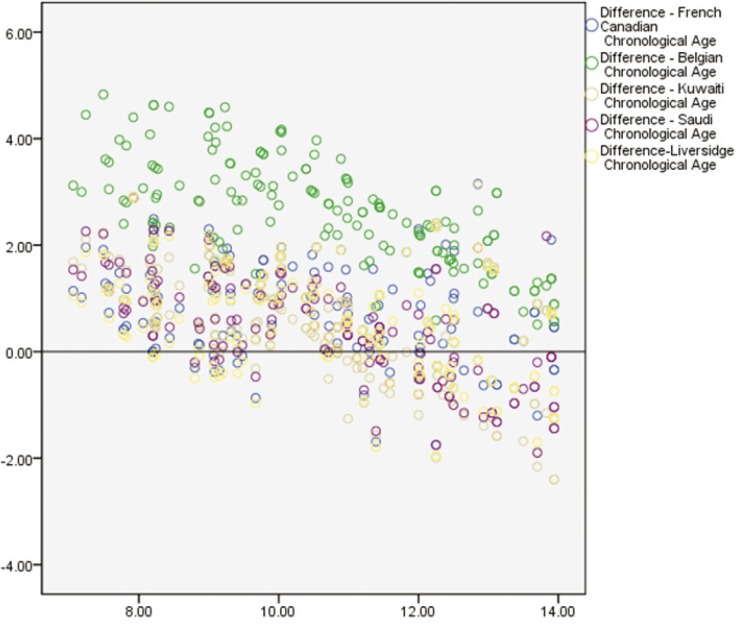
Estimation error compared to the chronological age of the current sample of children by the different methods.

The ICC was 0.994, indicating a high level of reproducibility. Applying the Demirjian method and Demirjian revised methods, the dental age (DA), chronological age (CA), and differences between DA and CA (DA-CA) for both genders and all age groups are presented in  Table 1. The paired t-test results for the total Demirjian method indicated that the mean CA was 8.94 (SD 2.74) [boys 9.27 (SD 2.73), girls 8.62 (SD 2.72)] and the mean DA ranged from minimum 8.89 (SD 2.46) [boys 9.23 (SD 2.47), girls 8.55 (SD 2.42)] using Baghdadi tables to maximum 10.98 (SD 3.46) [boys 11.19 (SD 3.45), girls 10.77 (SD 3.88)] using Belgian tables. The results indicated an over-aging of the sample as a whole by about 10 months using Demirjian tables, 5.5 months using Emran tables as well Kuwaiti tables, 24.7 months using Belgian tables, and 5 months using Liversidge international tables. The sample was under-aged by 0.6 month using Baghdadi tables. The overall discrepancies between CA and DA were statistically significant (*P* < 0.0001) for all methods with the exception of Baghdadi curves. Multinomial regression analyses for boys and girls using the DA as calculated by the different methods as predictors and CA as a constant found a close correlation between DA and CA indicated by a high R-squared value (boys 0.907, girls 0.926). The beta values (slope of the line) of Baghdadi method were closest to the study population for both boys and girls ( Table 2, Table 3). Figure 2 depicts the maturation curves of the study population using the Demirjian method and revised versions; generally, age prediction showed higher errors in younger ages and lower errors in older ages while at the same time Belgian tables showed the highest errors irrespective of age and gender cohorts (see also  Table 1). Figure 3 demonstrates estimation error compared to the chronological age of the current sample of children by the different methods.

**Table 1:**
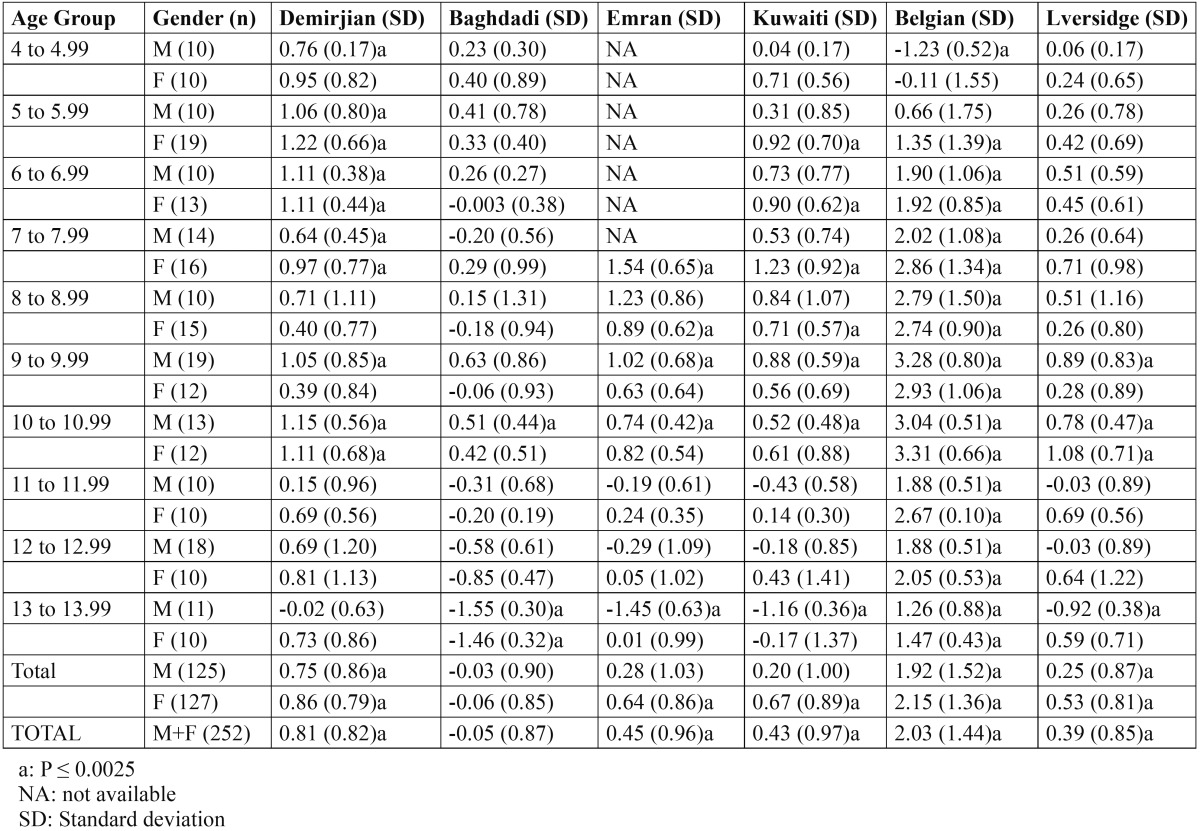
The mean differences between dental age and chronological age (DA – CA) according to different methods per age and gender cohorts.

**Table 2:**
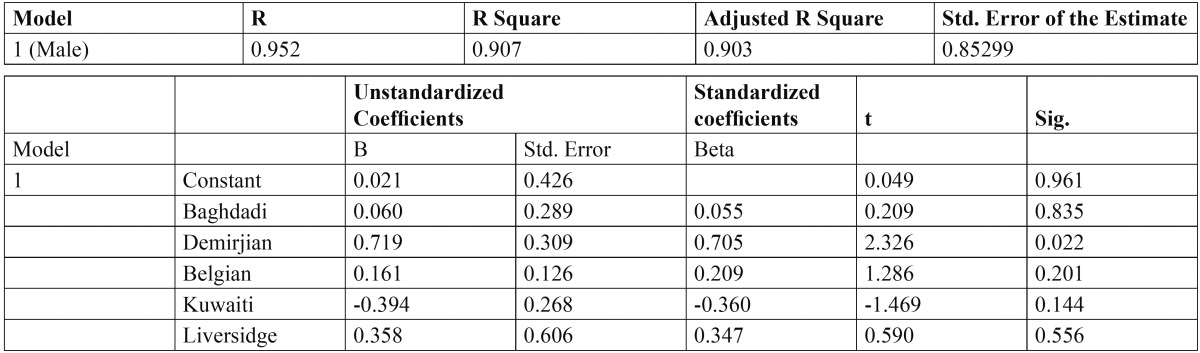
Cubic Regression Analysis for boys using the dental age as estimated by different methods as predictors and chronological age as a constant.

**Table 3:**
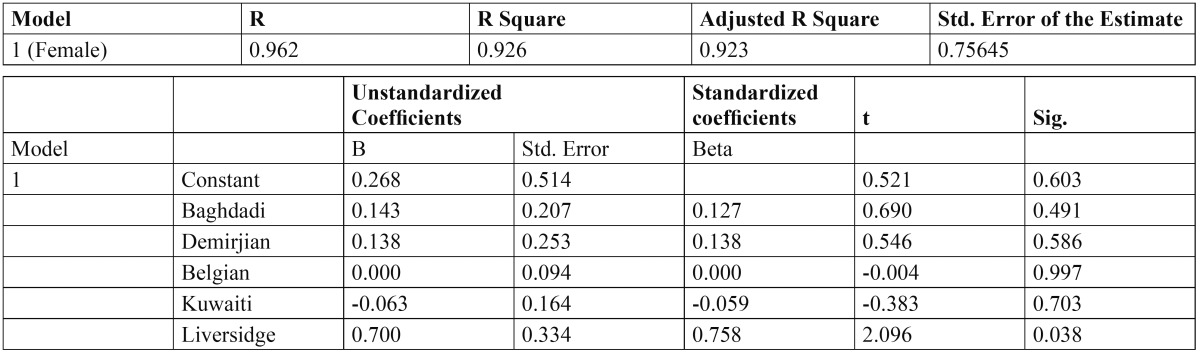
Cubic regression analysis for girls using the dental age as estimated by different methods as predictors and chronological age as a constant.

## Discussion

Accurate knowledge, or prediction, of age can be used to predict the optimal timing for treatment in orthodontic, orthopedic or pediatric clinical practice or to estimate chronological age of child skeletal remains in forensic or archeological contexts (14). From the several proposed methods developed for age determination, dental and skeletal development is considered the most reliable because of the low variability in dental maturation (14). Several methods have been proposed for assessing dental development, which is generally referred to as dental aging.

Dental aging comes in two forms: calcification (tooth development) and eruption patterns (15). Eruption refers to emergence of the tooth through the gum, rather than to emergence from the bone or to reaching the occlusal plane (16). This makes it impossible to use eruption for age estimation on skeletonized individuals in forensics. In addition, tooth emergence may be influenced by local exogenous factors, such as infection, obstruction, crowding, and premature extraction of the deciduous predecessor or adjacent permanent teeth (15). Most of the mentioned disadvantages can be avoided by using stages of tooth formation obtained from radiographical data on the calcification of teeth to determine dental maturity from in utero until late twenties, if the third molar is used.

The Demirjian eight-stage method is based on quantifying the dental development of seven lower permanent teeth between birth up to the age 17, when all the teeth’s apices, with the exception of third molars, presumed to be closed. Although the Demirjian method performs well in terms of observer agreement and correlation between estimated and true age (which is in agreement with the current study), the Demirjian original French-Canadian standards do not accurately estimate the chronological age in all the studied samples (4,6,17-22).

Generally, three scenarios were observed when applying the French-Canadian standards to several ethnicities. First, the Demirjian method overestimated aging compared with French-Canadian standards. This is in agreement with the findings for different European and worldwide populations (17-20). Most advanced overestimation was found by Koshy and Tandon in South Indian children: 3.04 and 2.82 yrs in boys and girls, respectively (23). Also, the results of current study in Saudi populations show similar overestimation but to lesser degree. Second, the Demirjian method underestimated aging which was reported by Moananui et al. in children of three ethnic groups living in New Zealand (24). Third, the Demirjian method accurately estimated aging. Burt et al.’s study on urban American children, aged 6 to 12, supports the appropriate use of Demirjian method on American population (15). Similar results were reported by Liversidge *et al.* who found no major differences in the timing of tooth formation stages, estimated using the Demirjian method, between children from Australia, Belgium, Canada, England, Finland, France, South Korea and Sweden (ages 2 to 16.99 years) (25).

Three reasons were stipulated to justify the differences in dental maturation found in many populations that could provide forensic anthropologists with erroneous results when calculating estimated age for the biological profile: ancestry or ethnicity, environment, and secular changes (8,9).

The Differences between real age and estimated age shown in several studies using a variety of methods have driven some authors to strive for more accurate results; therefore, new standards were developed (11-13). Two separate studies by Baghdadi and Pani and Baghdadi found that Demirjian method over-estimate age of Saudi boys and girls by about 10 months (10,26). Therefore, new age prediction models and maturation scores for Saudi population were developed based on the Demirjian method using multinomial (cubic) functions that re-quire validation (10). In an earlier study, Al Emran developed new maturation curves for Saudi boys and girls aged 8.5 years to 17 (8). The current study found that Saudi specific maturation curves are more accurate than original Demirjian curves. While Liversidge curves and Kuwaiti curves provided an acceptable way to estimate age, the Belgian curves should not be used to estimate Saudi age.

The vast majority of studies used t-test or ANOVA to examine the difference in dental maturity to the 50th per-centile of the reference method to a group of children expressed in terms of dental age. This method does not take into account known differences in developmental timings because, as pointed out by Liversidge (12), groups with significantly different average score can have similar maturity curves. This applies to analyses by both year cohorts and calculating a single value for the whole group. Regression analyses followed here allow studying age as a continuous variable rather than a grouped ordinal variable necessary while using the t-test or the ANOVA. Large samples are often recommended for growth studies. Flood et al. showed that smaller samples [n = 144] may be used when assessing dental maturity curves for forensic age estimation (1). Liversidege considered a sample of 10 or more boys or girls per year is representative (12). In the current study, panoramic radiographs for children younger than 4 years were not found due to ethical and practical considerations, making radiologic exposing of those at this age difficult and rarely scientifically justified. Children older than 14 years were not included in the sample because an earlier study found the Demirjian method inadequate for Saudi children older than 14 (10).

## Conclusions

After evaluation of the findings to the literature, it can be concluded that the Demirjian method has been shown to be modifiable to ensure that it is population specific. This paper tests population specific versions of the met-hod on the Saudi population with results that appear to show that the Saudi population method is most accurate on a Saudi population.
